# Use of Oral Tetracyclines in the Treatment of Adult Patients with Community-Acquired Bacterial Pneumonia: A Literature Review on the Often-Overlooked Antibiotic Class

**DOI:** 10.3390/antibiotics9120905

**Published:** 2020-12-14

**Authors:** Monique R. Bidell, Manjunath (Amit) P. Pai, Thomas P. Lodise

**Affiliations:** 1 Massachusetts General Hospital, Boston, MA 02114, USA; Monique.Bidell@MGH.HARVARD.EDU; 2 College of Pharmacy, University of Michigan, Ann Arbor, MI 48109, USA; amitpai@med.umich.edu; 3 Albany College of Pharmacy and Health Sciences, Albany, NY 12208, USA

**Keywords:** tetracycline, pneumonia, community-acquired pneumonia, doxycycline, minocycline, omadacycline

## Abstract

Oral tetracyclines have been used in clinical practice for over 60 years. Overall, one of the most common indications for use of oral tetracyclines is for treatment of adult outpatients with lower respiratory tract infections, including community-acquired pneumonia (CAP). Despite the longstanding use of oral tetracyclines, practice patterns indicate that they are often considered after other guideline-concordant oral CAP treatment options (namely macrolides, fluoroquinolones, and β-lactams). However, there are growing resistance or safety concerns with the available oral agents listed for outpatients with CAP in the updated American Thoracic Society (ATS)/Infectious Diseases Society of America (IDSA) CAP guidelines, especially among patients with comorbidities or notable risk factors for resistant pathogens. Given the need for alternative oral agents to macrolides, fluoroquinolones, and beta-lactams for adult outpatients with CAP, this review summarizes the literature on the use of oral tetracyclines (i.e., doxycycline, minocycline, and omadacycline) for this indication. As part of this review, we described their mechanism of action, common mechanisms of resistance, susceptibility profiles against common CAP pathogens, pharmacokinetics, pharmacodynamics, clinical data, and safety. The intent of the review is to highlight the important considerations when deciding between doxycycline, minocycline, and omadacycline for an adult outpatient with CAP in situations in which use of an oral tetracycline is warranted.

## 1. Introduction

Oral tetracyclines have been used in clinical practice for over 60 years [1]. These agents are characterized by their relatively broad activity against Gram-positive, Gram-negative, and atypical pathogens as well as a generally favorable safety profile [2,3,4,5,6]. One of the most common indications for use of an oral tetracycline-like antibiotic is for treatment of adult patients with community-acquired pneumonia (CAP). The updated American Thoracic Society (ATS)/Infectious Diseases Society of America (IDSA) CAP guidelines recommend use of doxycycline monotherapy as an option for outpatients without comorbidities or notable risk factors for resistant pathogens [7]. Doxycycline is also recommended for use in combination with a beta-lactam antibiotic for outpatient adult CAP patients with comorbidities and for hospitalized CAP patients who have contraindications to both macrolides and fluoroquinolones. Omadacycline, a derivative of minocycline and first-in-class aminomethylcycline antibiotic, was recently approved for the treatment of adult patients with community-acquired bacterial pneumonia (CABP) [8]. However, this agent was not included as a recommended treatment in the updated CAP guidelines as the guideline committee indicated that omadacycline required “further validation in the outpatient setting [7].” Minocycline was also not included in the updated CAP guideline despite its similarities to doxycycline. 

Despite the longstanding use of oral tetracyclines in the treatment of adult patients with CAP, practice patterns indicate that they are often considered after other guideline-concordant oral CAP treatment options (namely macrolides, fluoroquinolones, and β-lactams) [7]. However, there are high reported rates of *Streptococcus pneumoniae* resistance with some of the available oral agents listed for outpatients with CAP in the updated ATS/IDSA CAP guidelines. Macrolide resistance among *S. pneumoniae* is endemic in most areas throughout the world, limiting their use as monotherapy agents [9]. Pneumococcal resistance has also been reported to exceed 20% for the oral cephalosporins (i.e., cefpodoxime and cefuroxime) listed as first-line treatments for outpatients with comorbidities in the revised CAP guidelines [10,11,12]. In contrast to the macrolides and oral cephalosporins, amoxicillin and amoxicillin/clavulanate still have highly favorable in vitro activity against *S. pneumoniae* [13]. However, data indicate that standard dosing of these two agents is associated with suboptimal pharmacokinetic–pharmacodynamic (PK/PD) probability of target attainment profiles and is unlikely to provide adequate free plasma concentrations about the minimum inhibitory concentration (*f*T > MIC) for MIC values deemed susceptible by Clinical and Laboratory Standards Institute (CLSI) [14,15,16,17]. If one considers the PK-PD harmonized *S. pneumoniae* European Committee on Antimicrobial Susceptibility Testing (EUCAST) susceptibility breakpoint of ≤ 0.5 mcg/mL vs. the CLSI breakpoint of ≤ 2 mcg/mL, ~20% of isolates are non-susceptible to amoxicillin and amoxicillin/clavulanate [13,17,18]. More intensive dosing of these agents improves their probability of target attainment profiles but leads to a greater risk of adverse events and of disturbances in the intestinal microbiota [19]. Fortunately, fluoroquinolones still have excellent in vitro active against *S. pneumoniae* and there are no PK/PD dosing concerns. However, there have been increasing safety issues in recent years. The US Food and Drug Administration (FDA) and European Medicines Agency (EMA) have updated the labeling of all fluoroquinolones advising of the serious risk of multiple disabling and potentially irreversible adverse reactions associated with their use [20,21]. Most recently, the FDA updated the labeling of all fluoroquinolones to include the increased risk of aortic aneurysm rupture and aortic dissection [22,23].

Given the need for alternative agents to macrolides, fluoroquinolones, and oral beta-lactams with bioequivalent oral formulations for adult outpatients with CAP, this review summarizes the literature on the use of tetracyclines for this indication. As part of this review, we describe their mechanism of action, common mechanisms of resistance, susceptibility profiles against common CAP pathogens, pharmacokinetics, pharmacodynamics, clinical data, and safety. Given that the major unmet clinical need is for adult outpatients with CAP, only tetracycline agents with oral formulations are discussed (i.e., doxycycline, minocycline, and omadacycline) to inform their use in exclusively or upon transition to outpatient settings.

## 2. Methods

An extensive PubMed search was completed to acquire all the relevant literature on resistance mechanisms, susceptibility profiles, pharmacokinetics, pharmacodynamics, and clinical data pertaining to the use of doxycycline, minocycline, and omadacycline for the treatment of adult patients with CABP. Studies were reviewed for inclusion based on title and specific interest was placed on titles that included specific tetracycline agents, studies that specifically mentioned tetracyclines for CAP (e.g., clinical trials), or studies that compared resistance among CABP treatments for the most common bacterial pathogens (i.e., *S. pneumoniae*, *H. influenzae*, *M. catarrhalis*, *C. pneumoniae*, *M. pneumoniae*, *Legionella* spp.). Upon identification, all articles underwent cross-referencing to ensure that other relevant articles not captured in the initial search were reviewed and included as applicable. An emphasis was placed on surveillance studies published 2000 or later describing data from North America and Europe. We did not include *Staphylococcus aureus* in our review given that it is an infrequent cause of CAP in outpatient treatment settings and typically results in a more severe pneumonia that requires hospitalization [13].

## 3. Mechanisms of Action and Resistance

The tetracycline class of antibiotics [24] inhibit bacterial protein synthesis via inhibition of aminoacyl-tRNA to the A site of the 30S ribosomal subunit. This prevents addition of new amino acids to the developing peptide chain [1,25]. The four main mechanisms of resistance to traditional tetracycline antibiotics can be characterized as active efflux, presence of ribosomal protection proteins, enzymatic deactivation, and target modifications (albeit rare) [26,27]. Resistance to tetracyclines largely involves *tet* genes that encode for various Tet proteins. Efflux pumps expel tetracyclines to the periplasm, decreasing intracellular concentrations available for ribosomal binding [1]. Tet(K) and Tet(L) are the most well-described efflux pumps in Gram-positive bacteria, while Tet(A) and Tet(B) are more common in Gram-negative bacteria [28,29]. Ribosomal protection proteins (RPPs) serve to “rescue” inhibited ribosomes and restore protein synthesis capability [26]. This is achieved by catalyzing GTP-dependent release of tetracycline from the 30S subunit via introducing ribosomal conformational changes [1,2,3]. The most well-described RPPs are Tet(O) and Tet(M) [1]. Less commonly, resistance can also be conferred by “inactivating” enzymes that modify the tetracycline core structure. The most well-characterized tetracycline modifying enzyme is Tet(X) [1,26].

Resistance to tetracycline, doxycycline, minocycline and omadacycline varies depending on the expressed mechanism. Tet(K) and Tet(L) efflux pumps in gram positive bacteria exhibit affinity for tetracycline and doxycycline, resulting in isolates with higher MIC values [1,29]. Tet(B) efflux pumps expressed primarily by gram negative pathogens effectively expel tetracycline, minocycline, and doxycycline [1]. The number of pumps expressed, as well as other bacterial factors, appears to affect the degree of agent non-susceptibility [1]. The RPP Tet(M) exhibits similar a phenotype as Tet(B) in that it also confers resistance to tetracycline, minocycline, and doxycycline [30]. Omadacycline is not affected by either of these characteristic tetracycline resistance mechanisms, seemingly due to its bulky C-9 side chain group. This structural addition makes omadacycline a poor efflux pump substrate and also creates steric hindrance to block RPPs from their ribosomal binding site [1,24,26]. The inactivating enzyme Tet(X) is capable of conferring resistance to all commercially available tetracyclines including omadacycline via addition of a hydroxyl group to the C11 position between the B and C rings of the tetracycline core structure [1,2,26]. However, expression of this mechanism appears to be uncommon. Omadacycline activity can also be impaired in the setting of rRNA mutations that can reduce its affinity for the ribosomal binding site [2], though frank resistance may not occur. These rRNA mutations also appear to confer a fitness cost resulting in impaired bacterial growth [24].

With respect to common bacterial CAP pathogens, tetracycline resistance has been well-described for *S. pneumoniae.* The most reported mechanism of resistance is expression of the RPP Tet(M) [25,29,31]. Notably, the Tn916 family transposons in pneumococci harbor the erm(B)-carrying mobile genetic elements, which confers resistance to the macrolides, and many Tn916 derivatives also carry *tet*(M). Therefore, cross-resistance between macrolides and doxycycline/minocycline may be observed [32]. With respect to other CABP pathogens, *Mycoplasma* spp. and *Haemophilus* spp. can also express *tet*(M), while *Moraxella catarrhalis* has been reported to express *tet*(B) [29]. Other resistance mechanisms for these species as well as other CABP pathogens have not been well studied [29].

## 4. In Vitro Activity

Current CLSI susceptibility breakpoints for tetracycline and doxycycline against *S. pneumoniae* are ≤ 1 mcg/mL and ≤ 0.25 mcg/mL, respectively (zone diameters ≥ 28 mm for both), while the FDA-assigned omadacycline susceptibility breakpoint is ≤ 0.12 mcg/mL [33]. The *S. pneumoniae* CLSI breakpoints for tetracycline were updated in 2013 (previously ≤ 2/4/≥ 8 mcg/mL for susceptible, intermediate and resistant, respectively), which is also when doxycycline-specific breakpoints were established [34]. Tetracycline breakpoint revision was prompted by data demonstrating that the previous breakpoints did not sufficiently differentiate between isolates that expressed *tet* genes from those that were wild-type [34]. In one study, the *tet*(M) gene was detected in 43% (three of seven) of strains with a tetracycline MIC at the prior breakpoint of 2 mcg/mL. Doxycycline MICs in the seven *tet*(M) positive strains ranged from 0.25–1 mcg/mL [34]. Tetracycline and omadacycline susceptibility breakpoints for *H. influenzae* are the same at ≤ 2 mcg/mL [33]. With respect to routine in vitro susceptibility testing, tetracycline susceptibilities are most frequently performed and reported. Tetracycline susceptibility can be used as a reliable surrogate for doxycycline and minocycline susceptibility with >95% accuracy [34]. However, due to variable resistance mechanisms discussed previously, tetracycline resistance does not necessarily inform doxycycline or minocycline susceptibilities [3]. These principles also hold true for omadacycline.

In vitro data (MIC_50_, MIC_90_) for tetracycline, doxycycline, minocycline, and omadacycline against CABP pathogens from observational cohort and large surveillance studies over the last approximately 20 years are summarized in Table 1. A focus was placed on data from North America or Europe. Non-susceptibility rates are also included when available, though they are subject to the susceptibility breakpoints at the time of the respective publication. As mentioned previously, tetracycline resistance may be underestimated in studies using pre-2013 CLSI breakpoints. 

## 5. *S. pneumoniae*

Tetracycline MIC data are widely reported for *S. pneumoniae*, with several surveillance studies describing non-susceptibility rates of approximately 15 to 30% [12,37,39]. Doxycycline and minocycline susceptibility data is not widely reported in large surveillance analyses. Data from three moderately sized studies (*n* = 1179 to 2313) reported doxycycline non-susceptibility rates of approximately 25% among *S. pneumoniae* [34,40,50]. The highest reported rate was 41% from one small analysis of 29 isolates (*n* = 29) [43]. Notably, non-susceptibility rates appear to be higher among penicillin-resistant isolates, with three studies reporting rates of about 60% [40,43,50]. One surveillance study of US and European isolates described doxycycline resistance rates with respect to penicillin susceptibility, observing rates of 2.9% among penicillin-susceptible isolates (*n* = 661) and 64.2% among penicillin-resistant strains (*n* = 260) [50]. Among 115 ceftriaxone non-susceptible isolates from the same study, doxycycline resistance frequency was noted to be 83.5% [50]. Omadacycline non-susceptibility rates against *S. pneumoniae* have been described across a number of recent studies. Non-susceptibility is reported infrequently (<3%) [13,42,44,45,50] regardless of penicillin or tetracycline susceptibility [13,44,50].

Very few studies have directly compared MIC or susceptibility data across multiple tetracycline agents. In one study by Macone et al., MIC_90_ values for tetracycline, doxycycline, minocycline and omadacycline among 41 *S. pneumoniae* isolates were 32, 4, 8, and 0.125 mcg/mL, respectively. MICs were further stratified with respect to expression of the *tet*(M) gene. In isolates lacking *tet*(M), MICs were consistently low for tetracycline, doxycycline, minocycline, and omadacycline (≤0.06–0.25 mcg/mL). However, among the 22 isolates that harbored *tet*(M), respective tetracycline, doxycycline, and omadacycline MICs ranged from 4–64 mcg/mL, 2–4 mcg/mL, and ≤0.06–0.25 mcg/mL. Omadacycline was also noted to retain activity against isolates that were collectively resistant to tetracycline, penicillin and azithromycin [41].

## 6. *Haemophilus* spp., *M. catarrhalis*, and Atypical Pathogens

Data suggest that tetracycline and omadacycline non-susceptibility rates against *H. influenzae* and *M. catarrhalis* are low. In one study examining isolates from 2015–2017, 1.4% (*n* = 1172) of *H. influenzae* and 0.7% (*n* = 613) of *M. catarrhalis* were tetracycline-resistant [39]. Against *H. influenzae*, omadacycline was the most active (MIC_90_ of 2 mcg/mL), followed by doxycycline (MIC_90_ of 4 mcg/mL) and tetracycline (MIC_90_ of 32 mcg/mL) [41]. Activity was not affected by the presence or absence of β-lactamase [13,45]. Few data are available on susceptibility among atypical pathogens. Waites et al. described MIC_90_ values for tetracycline, doxycycline, and omadacycline among a small number of *M. pneumoniae* isolates to be 0.5 mcg/mL, 0.5 mcg/mL, and 0.25 mcg/mL, respectively [47]. One study reported similarly low MIC_90_ values for doxycycline (0.125 mcg/mL) and omadacycline (0.25 mcg/mL) against *C. pneumoniae* [46]. Limited data are available for *Legionella* spp., though one study described MIC_90_ values of 1 mcg/mL for doxycycline and 0.25 mcg/mL for omadacycline [49]. 

## 7. Pharmacokinetics

Recommended dosing regimens of doxycycline, minocycline, and omadacycline for adult outpatients with CABP are shown in Table 2. Because all have similarly long serum elimination half-lives, in the range of 16–24 h (Table 3, adapted from Rodvold et al., Clinical Pharmacokinetics (2020) 59:409–425), loading doses are included in the product labeling for omadacycline and encouraged for doxycycline and minocycline when used for adult patients with serious infections like CABP [51,52]. Notably, the product labeling of omadacycline indicates that an intravenous (IV) loading dose should be initiated on day 1 for adult patients with CABP, followed by either IV or oral (PO) daily maintenance doses. 

There are several distinguishing PK features between these agents. Both doxycycline and minocycline exhibit 90–100% oral bioavailability [58], and therefore IV and oral doses are bioequivalent. There are only modest reductions in absorption (20% for doxycycline [56] and minimal for minocycline [71]) when they are taken with food. However, more substantial reductions in bioavailability can be expected upon co-administration with divalent and trivalent cations as well as bismuth subsalicylate due to chelation [51,53,54]. In contrast, the bioavailability of omadacycline is reported to be approximately 35% [72,73,74]. To compensate for the lower absorption and ensure therapeutic equivalent systemic exposures between the IV and oral formulation, the oral maintenance dose (i.e., 300 mg) is three times the 100 mg IV dose [8]. There appears to be a greater food effect observed with omadacycline [74] relative to doxycycline and minocycline. Patients are required to fast for at least 4 h prior to taking oral omadacycline. After oral omadacycline dosing, no food or drink (except water) is to be consumed for 2 h and no dairy products, antacids, or multivitamins for 4 h [8].

There are also distinctive differences in observed plasma AUC values and apparent clearances between agents, with higher AUC values and lower clearances reported for doxycycline and minocycline relative to omadacycline (Table 3) [52]. For doxycycline and minocycline, the average AUCs for 200 mg/day oral doses range from 85–108 mg·h/L and 68.6–71.3 mg h/L, respectively. In contrast, the daily AUC for omadacycline for 300 mg oral or 100 mg IV dose is ~8–10 mg h/L. However, the free plasma AUC are likely similar for agents as doxycycline and minocycline exhibit 75–90% protein binding [67,75] in serum while omadacycline is only 20% [72]. Though the implications of this difference for treatment of patients with CAP in clinical practice is unclear, it is well established that unbound or free drug is microbiologically active and penetration into the infection site often varies as a function or extent of protein binding [76].

Currently, there are only quality PK data on lung penetration for omadacycline [77]. In a 41-subject healthy volunteer study, the 24 h area under the curve (AUC_24_) values in epithelial lining fluid (ELF) and alveolar lung macrophages (AM) were 17.2 and 302.5 mg h/L, respectively. The mean AUC_24_ ratios of ELF and AM to total plasma were 1.5 and 25.8, respectively. Two studies have quantified the ability of doxycycline to concentrate in sputum and lung tissue. In an assessment based on single time point estimations, doxycycline penetration into sputum is 8–28%, estimated over 16 h [78]. In a single time point lung penetration estimation study, the overall serum to tissue ratio in lungs for doxycycline was estimated to be 0.68 [79]. In a study of 14 patients undergoing lung surgery who received minocycline, the mean lung tissue to plasma and sputum to serum ratio ratios were 3.71 +/− 2.36 and 0.56 +/− 0.47, respectively [80]. 

There are several pharmacokinetic features common to doxycycline, minocycline, and omadacycline. These agents are primarily eliminated unchanged by the renal and biliary routes, with the exception of minocycline, which undergoes some hepatic metabolism [81]. Dose adjustments for weight, age, gender, race, renal impairment, or hepatic dysfunction are not required for these agents [28,58,60,64,69,77,82,83,84,85,86,87]. There are high quality PK data in specialized population to support these recommendations with omadacycline [69,77,82,83], and to a lesser extent, doxycycline [28,58,60,64,84,85,86,87]. There is less information on the PK of minocycline in specialized populations [51,60,85,86] although one would not anticipate any clinically meaningful differences given the structural similarities between this agent and doxycycline. However, the FDA product labeling for minocycline indicates that current data are insufficient to determine if dose adjustments are warranted among patients with renal impairment [54]. 

None of the discussed tetracycline agents are expected to interact with drugs metabolized by cytochrome P450 enzymes, thereby limiting the potential for clinically significant cytochrome P450 drug–drug interactions. However, barbiturates, carbamazepine, rifamycins, and phenytoin have been shown to decrease the half-life of doxycycline, suggesting that it may undergo some hepatic metabolism [53,88]. Patients who are on vitamin K antagonist anticoagulant therapy may require lower doses in the setting of concurrent tetracycline use as these agents have been shown to depress plasma prothrombin activity. Use of tetracyclines may also render oral contraceptives less effective [53,54]. 

## 8. PK/PD Infection Model Studies

Studies quantifying the PK/PD profile of these agents against CABP pathogens have been largely limited to omadacycline. In a one-compartment in vitro infection model of *H. influenzae* (*n* = 5 strains with MIC values of 1–2 mg/L) that was designed to mimic conditions in the ELF, the AUC_24_/MIC ratios required for 1-log_10_ and 2-log_10_ reductions in bacterial load were 8.91 and 11.1, respectively [89]. In a neutropenic mouse pneumonia model of *S. pneumoniae* (*n* = 4 strains with MIC values of 0.0315–0.125 mg/L) [90], both the unbound plasma and ELF AUC_24_/MIC ratios had high correlations with efficacy. The required ELF AUC_24_/MIC for bacterial killing varied across different strains, with median ratio values of 15.5 and 23.2 for 1-log_10_ and 2-log_10_ reductions in bacterial load, respectively. Not surprisingly, the observed unbound plasma AUC_24_/MIC and ELF AUC_24_/MIC ratios were largely similar, as the penetration of omadacycline from plasma into ELF approached 100%. Utilizing the median ELF AUC_24_/MIC ratio targets identified in this study and the population predicted ELF AUCs in patients at near steady state conditions [77], standard IV and oral dosing of omadacycline is expected to produce efficacy against most *S. pneumoniae* and *H. influenzae* strains, including those with tetracycline resistance [13]. 

There are scant data on the use of in vitro pharmacokinetic or animal pharmacokinetic/pharmacodynamic infection models to describe the antibacterial effects of doxycycline and minocycline against common CABP pathogens. The best available data for doxycycline come from a neutropenic mouse infection model study of four *S. pneumoniae* isolates [91]. Christianson et al. demonstrated that a free plasma AUC_24_/MIC target of 24 for doxycycline was associated with net stasis and that a value of 120 was associated with a 2-log10 kill. Although it is difficult to compare across infection model studies, the free plasma AUC_24_/MIC ratios for doxycycline were considerably higher than those observed for omadacycline in the neutropenic mouse pneumonia infection model study of *S. pneumoniae.* No data on the PK/PD profile of minocycline in pre-clinical PK/PD infection model studies of CABP pathogens are currently available.

## 9. Clinical Studies

A limited number of randomized clinical trials have evaluated these agents for the treatment of adult patients with suspected or documented CAP (Table 4). To date, six randomized comparator CAP clinical trials have included doxycycline and one has included omadacycline [92,93,94,95,96,97,98]. We are unaware of any randomized clinical trial that has evaluated minocycline for the treatment of adult patients with CAP. While several observational studies describe the use of minocycline for CAP, all but one report [99] involve treatment of *M. pneumoniae* [100,101,102,103,104]. The one non-*M. pneumoniae*-focused study included patients with mixed pneumonia presentations, only 14 of which received IV minocycline for CAP. 

Overall, the results of the doxycycline randomized clinical trials were largely positive, but most were limited in sample size, of varying quality, and performed prior to the year 2000. The first double bind randomized trial of adult patients with lower respiratory tract infections including CAP was conducted by Harazim and colleagues [92] that compared oral doxycycline to oral ofloxacin. Of the 230 enrolled patients in the trial, 131 had CAP and were evaluable. A satisfactory clinical response was observed in 90.0% of patients in the doxycycline group vs. 96.8% of patients in the ofloxacin group (*p*-value not provided). More doxycycline-treated CAP patients failed relative to ofloxacin-treated CAP patients (seven patients vs. two patients, respectively). Overall, regardless of type of lower respiratory tract infection type, seven patients enrolled in the doxycycline group withdrew due to adverse events vs. one patient in the ofloxacin group. In a similarly designed double blind, randomized trial of adult patients with lower respiratory tract infections (104 with CAP) [93], oral doxycycline was compared to the oral macrolide spiramycin. Sparse details on the study findings are available, but no differences in clinical cure between treatment groups among CAP patients were noted (overall clinical cure reported as 84%). In the entire study population, side effects occurred in 21.4% of patients in the doxycycline group vs. 22.5% of patients in the spiramycin group. Most side effects in each group were gastrointestinal in nature. Only one patient in the doxycycline group withdrew from the study secondary to feeling unwell and blurred vision. In another double blind randomized trial of patients with lower respiratory tract infections that included 25 patients with CAP [94], oral doxycycline was compared to erythromycin acistrate. Though specific details regarding patients with CAP were not provided, overall clinical response exceeded 96% in both treatment groups, and a similar number of patients in each group reported side effects that were predominantly gastrointestinal in nature. 

The largest randomized CAP trial that included doxycycline was performed by the Nordic Atypical Pneumonia Study group [95]. This study compared oral doxycycline to oral fleroxacin, a quinolone with limited activity against *S. pneumoniae*, among adult inpatients and outpatients with CAP. Of the 411 patients enrolled in this study, an etiology was confirmed in 270 (66%) cases. Nearly half of the confirmed cases involved *Mycoplasma* spp., *Chlamydia* spp., or *Legionella* spp. (*n* = 133) and many had a mixed etiology (*n* = 93). In the intention-to-treat analyses, clinical response rates in doxycycline- vs. fleroxacin-treated patients were 93% vs. 86%, respectively, at the first follow up visit and 85% vs. 75% (difference 10%, lower bound for one-sided 95% confidence interval—17.3%) at the second follow up visit. Higher response rates were observed with doxycycline relative to fleroxacin among patients with CAP due to *S. pneumoniae* and/or *Mycoplasma* spp. In an unblinded randomized clinical trial of hospitalized adult patients with mild-to-moderately severe CAP [96], empiric intravenous doxycycline was compared with other routinely used antibiotic regimens selected at the treating physicians’ discretion. Although it is difficult to draw definitive conclusions from this study given the limited sample size (*n* = 87) and lack of details on the treatments received in the comparator group, median time to clinical response and hospital stay were significantly shorter in the doxycycline group relative to the comparator group (2.2 +/− 2.6 days vs. 3.8 +/− 6.4 days, *p* = 0.001). Similarly, in another small double-blinded, randomized trial of adults hospitalized patients with CAP (*n* = 66), the mean hospital length of stay was significantly shorter for patients randomized to receive doxycycline (IV or PO) relative to levofloxacin (IV or PO) (4.0 +/− 1.8 days vs. 5.7 +/− 2.1 days, *p* < 0.0012) without a difference in clinical response (*p* = 0.84) [97]. However, the authors attributed the hospital length of stay difference to the small sample size and not decreased efficacy of levofloxacin. Beyond these randomized clinical trials, observational studies have reported that oral doxycycline (*n* = 22) resulted in similar outcomes (i.e., duration of fever after treatment initiation and side effects) relative to azithromycin (*n* = 83) for the treatment of pneumonia caused by *Chlamydia* spp. (*p* > 0.05) [105]. As part of the Australian CAP study, beta-lactam plus doxycycline was also found to result in similar outcomes as beta-lactam plus macrolide therapy among adult patients with non-severe CAP due to either atypical or typical pathogens [106]. 

The most rigorous study evaluating tetracycline-like agents for adult patients with CAP is OPTIC [98], a multi-national Phase 3 randomized, double-blind, double-dummy, non-inferiority trial that compared omadacycline to moxifloxacin. In OPTIC, adult patients with CABP (Pneumonia Severity Index risk classes II–IV) were randomized to receive omadacycline 100 mg IV every 12 h for two doses, then 100 mg intravenously every 24 h, or moxifloxacin 400 mg intravenously every 24 h in a 1:1 ratio. A transition to oral omadacycline 300 mg every 24 h or moxifloxacin 400 mg every 24 h was allowed after 3 days, and the total treatment duration was 7 to 14 days. In the intention-to-treat population, omadacycline was noninferior to moxifloxacin with regard to early clinical response (81.1% and 82.7%, respectively; difference, −1.6 percentage points; 95% confidence interval (CI), −7.1 to 3.8), defined as survival with improvement in two of the four primary pneumonia symptoms with no worsening in symptoms at 72–120 h after the initial dose. Investigator-assessed clinical response rates at the post-treatment evaluation were also found to be similar between omadacycline- and moxifloxacin-treated patients (87.6% and 85.1%, respectively; difference, 2.5 percentage points; 95% CI, −2.4 to 7.4). The incidence of adverse events was also similar between treatment groups (41.1% in the omadacycline group vs. 48.5% in the moxifloxacin group) apart from *Clostridioides difficile* infections (CDI), which occurred in eight patients (2%) who received moxifloxacin and no patients who received omadacycline. A mortality imbalance was observed in the trial, with eight deaths (2%) occurring in the omadacycline group and four deaths (1%) in the moxifloxacin group. All deaths occurred in patients over 65 years of age and most had multiple comorbidities. Although the cause of the mortality imbalance has not been established and mortality rates were consistent with other modern CABP trials [107,108], a warning was added to the package insert of omadacycline recommending close monitoring of CABP patients, particularly in those at higher risk for mortality [8]. 

## 10. Safety

The reported incidence of adverse events with use of tetracycline agents is low [3,109]. Side effects common to the class include gastrointestinal disturbances, esophagitis, photosensitivity, pigmentation changes, pediatric tooth discoloration, central nervous system effects (e.g., lightheadedness, dizziness) and rarely, anti-anabolic action, pseudotumor cerebri, hepatotoxicity, hypersensitivity, and idiopathic intracranial hypertension [109]. Although comparative studies are lacking, there does appear to be some differences in their side effect profiles, especially between doxycycline and minocycline [3,109]. In an assessment of reported adverse events for doxycycline and minocycline using data from the MedWatch Adverse Event Report program, clinical trials, and case reports, doxycycline had fewer reported adverse events despite being prescribed three times as often as minocycline [109]. Gastrointestinal adverse events were most common with doxycycline while dizziness, lightheadedness, and gastrointestinal effects were most common with minocycline use. Minocycline is also more likely to cause other central nervous system effects (i.e., dizziness, lack of concentration, ataxia, vertigo, tinnitus associated with weakness, nausea and vomiting) and pigmentation of various body sites [3].

Data regarding the safety of omadacycline has largely been limited to clinical trials [98,110,111,112] and pharmacokinetic studies [2,30,69,70,72,74,77,82,83], as scant real-world usage data are currently available. Overall, the safety profile of omadacycline to date is consistent with other oral tetracyclines. In the OPTIC pneumonia study, adverse events with omadacycline were low and comparable to those associated with moxifloxacin [98]. The most frequently occurring adverse events with omadacycline across its clinical studies were nausea and vomiting; however, rates were low (2.4% and 2.6%, respectively) in the OPTIC study [98]. Pooled safety data from omadacycline’s two OASIS acute bacterial skin and skin structure infection studies showed higher nausea and vomiting rates [110,112]. This was largely driven by high rates of nausea (30.2%) and vomiting (16.2%) reported with omadacycline in its oral-only ABSSSI phase 3 clinical trial (OASIS-2) [110]. In OASIS-2, nausea in patients taking omadacycline was mild to moderate and mostly limited to the first two days of therapy when the oral loading dose of 450 mg per day was administered. However, only one patient in omadacycline group from both OASIS-1 (0.3%) and OASIS-2 (0.3%) discontinued treatment due to nausea and vomiting. 

Although all antibiotics have the potential to cause CDI, tetracyclines have been associated with a lower risk relative to other commonly used CAP antibiotics and may actually protect against infection [113]. In a meta-analysis of antibiotics and the risk of community-associated CDI infection, tetracyclines had no effect on CDI risk while the fluoroquinolones, cephalosporins, carbapenems, monobactams, and clindamycin had the highest effects, followed by the penicillins, macrolides, and sulfa antibiotics [114]. As mentioned previously, CDI occurred in 8/388 patients (2%) who received moxifloxacin and 0/382 patients (0%) who received omadacycline in the recent OPTIC study [98]. No cases of CDI were observed in the two phase 3 OASIS studies of omadacycline in patients with ABSSSIs (OASIS-1 and OASIS-2) [110,112]. Although the exact reason for the protective CDI effects remains to be clarified, the most straightforward explanation is because of their in vitro activity against anaerobic bacteria, including *C. difficile* [115,116]. Furthermore, tetracyclines primarily undergo absorption in the upper gastrointestinal tract, which may cause less disruption of the gut microbiota and thus a lower potential to incite CDI infection [117]. Lastly, as a protein synthesis inhibitor, the lower CDI risk may also be due to attenuation of *C. difficile* toxin production. 

## 11. Discussion

The collective findings from this review suggest that doxycycline, minocycline, and omadacycline are viable options for the treatment of adult outpatients with CABP. Although currently available data do not support routine prioritization of doxycycline, minocycline, or omadacycline over other oral adult outpatient CAP therapies, these agents potentially should be considered as preferred treatment options when there are resistance or safety concerns with other the CAP guideline concordant oral therapies. It also may be prudent to consider them as one of the first-line agents in adult CAP outpatients at an elevated risk for CDI, given that tetracyclines have been associated with a substantially lower risk of infection relative to other commonly used CAP antibiotics [113,114]. They also may be a preferred treatment option in patients with contraindications to β-lactams and in patients who are at risk for adverse cytochrome P450 interactions. 

Despite the potential advantages to use of a tetracycline agent for treatment of outpatient CAP, there are several important considerations when deciding between doxycycline, minocycline, and omadacycline. While doxycycline has been a longstanding oral tetracycline used to treat CAP in adult patients, there are potential resistance concerns with its use, particularly as monotherapy. Local susceptibility data should guide empiric use of doxycycline whenever possible. Recent surveillance data indicate that doxycycline non-susceptibility rates among *S. pneumoniae* respiratory isolates often exceeds 25% but is variable by region [10]. In the revised CAP guidelines, macrolide monotherapy is discouraged if local pneumococcal resistance is ≥ 25% [7]. If this same criteria for use are applied to doxycycline, empiric doxycycline monotherapy should be avoided unless regional pneumococcal resistance rates to this agent are known. Use of doxycycline in combination with a β-lactam is an alternative option, but there are scant efficacy and clinical data to support the use of β-lactam and doxycycline combination therapy [106], especially in the presence of pneumococcal resistance to doxycycline. 

Omadacycline is a reasonable monotherapy option when use of an oral tetracycline is warranted for an adult outpatient with CAP, particularly when there is concern for pneumococcal resistance to doxycycline, macrolides, amoxicillin, amoxicillin/clavulanate, and oral cephalosporins. Surveillance studies indicate that omadacycline retains in vitro activity against *S. pneumoniae* strains that are doxycycline-resistant [41]. Although the revised CAP guidelines did not endorse the use of omadacycline for outpatients with CAP due to limited data [7], it is important to note that omadacycline is the only tetracycline with therapeutically equivalent IV and oral formulations that has satisfied modern FDA regulatory approval requirements and demonstrated non-inferiority to a fluoroquinolone for the treatment of adult CABP patients in a contemporary Phase 3 clinical trial [98]. Notably, omadacycline is currently only available in the US and its current product labeling indicates that IV therapy is required on day 1 of treatment, with the option to switch to oral therapy thereafter. The requirement of initial IV therapy potentially limits the initial site of care options for omadacycline. However, many adult outpatients with CAP in the US present to hospital emergency departments [118] for treatment, where IV administration would be feasible. Furthermore, use of IV omadacycline therapy on day 1 of treatment may soon no longer be necessary, as a supplemental New Drug Application (NDA) to support an oral-only dosing label for CABP was recently submitted to the FDA. The supplemental NDA was based in part on the results of an oral-only omadacycline PK study that was completed in adults with CABP [119]. It is also important to note, as with any new outpatient antibiotic, there is the potential for issues with drug access, prescription coverage, and out-of-pocket patient expenses with omadacycline. 

Lastly, there are limited clinical data supporting the use of minocycline for the treatment of adult outpatients with CAP. Although it was outside the scope of this review, the major antibiotic stewardship advantages of minocycline are related to its enhanced antimicrobial activity against resistant Gram-negative pathogens, including *Acinetobacter* spp., *Stenotrophomonas* spp. and extended spectrum β-lactamase producing Enterobacterales. Given available data, it appears most prudent to consider minocycline for treatment of adult outpatients with CAP when doxycycline is unavailable (i.e., drug shortage) or there is a need for concurrent activity against highly resistant Gram-negative pathogens [120].

In conclusion, there are growing pneumococcal resistance or safety concerns among the array of oral agents recommended in the updated ATS/IDSA guidelines for treatment outpatients with CAP. Data suggest that doxycycline, minocycline, and omadacycline are viable options for the treatment of adult outpatients with CAP when there are resistance, safety, or CDI concerns with other the CAP guideline concordant oral therapies. Doxycycline has been a longstanding oral tetracycline for adult outpatients with CAP, but its empiric use as monotherapy is limited by potential pneumococcal resistance concerns. Minocycline’s PK profile and available in vitro susceptibility data suggest that it would be similarly effective as doxycycline for adult outpatients with CABP, though lack of clinical data precludes its routine use. When a monotherapy tetracycline agent is desired, omadacycline is a reasonable option. However, omadacycline has only been evaluated in one phase 3 CABP clinical trial to date and real-world effectiveness and safety data are scant. Lastly, more comparative effectiveness studies are needed to support oral omadacycline and doxycycline, both as monotherapy and in combination with a β-lactam, for treatment of adult outpatients with CAP.

## Figures and Tables

**Table 1:** Minimum inhibitory concentrations and non-susceptibility rates for oral tetracyclines against CAP pathogens *.

Pathogen	Tetracycline MIC_50_/MIC_90_/%NS	Doxycycline MIC_50_/MIC_90_/%NS	Minocycline MIC_50_/MIC_90_/%NS	Omadacycline MIC_50_/MIC_90_/%NS	Reference (Geographic Location, Years Obtained)
*S.pneumoniae*	0.5/≥32/15.6% (*n* = 1817)				Doern 2005 (US; 2002–2003) [10]
	≤4/>8/14.2% (*n* = 2110)				Pottumarthy 2005 (North America; 2002–2003) [11]
	-/>8/14.4% (*n* = 9296)				Sahm 2008 (USA; 2001–2005) [12]
	-/-/17% (*n* = 75)				Desai 2010 (US; 1994–2004) [35]
	≤4/>8/19.5% (*n* = 246)				Hoban 2003 (US and Canada; 2000) [36]
	0.25/≥8/14.6–15.9% (*n* = 39,495)				Jenkins 2008 (USA; 2000–2004) [37]
	-/16/14.9% (*n* = 1300)				Melo Cristino 2013 (Portugal; 2003–2004) [38]
	0.5/>4/30.2% (*n* = 1736)				Sader 2018 (Europe, Asia, Latin America; 2015–2017) [39]
	0.5/>8/26.7% (*n* = 2313)	0.25/8/26.8% (*n* = 2313)			Jones 2013 (North America, Europe, Latin America, Asia-Pacific; 2010) [34]
	2/>8/23.8% (*n* = 1443)	0.5/>4/23.1% (*n* = 1443)			Jones 2004 (USA; 1999–2002) [40]
	16/32/- (*n* = 41)	2/4/- (*n* = 41)	2/8/- (*n* = 41)	≤0.06/0.125/- (*n* = 41)	Macone 2014 (USA) [41]
	0.5/>8/23.1% (*n* = 1179)	0.25/8/23.6% (*n* = 1179)		0.06/0.06/-	Pfaller 2018 (USA and Europe; 2014) [42]
		-/-/41% (*n* = 29)			Lederman 2003 (USA; 2001–2002) [43]
				0.06/0.12/1.1% (*n* = 3153)	Pfaller 2020 (USA and Europe; 2016–2018) [13]
				0.06/0.12/0.3% (*n* = 1314)	Pfaller 2018 (USA and Europe; 2016) [42]
				0.06/0.12/1.4% (*n* = 968)	Huband 2019 (USA and Europe; 2017) [44]
				0.06/0.06/1.8% (*n* = 6253)	Pfaller 2017 (North America, Europe, Asia-Pacific, Latin America; 2010–2011) [45]
Penicillin-resistant *S.pneumoniae*	-/-/44.3% (*n* = 348)				Pottumarthy 2005 (North America; 2002–2003) [11]
	>4/>4/95.2% (*n* = 455)				Sader 2018 (Europe, Asia, Latin America; 2015–2017) [39]
		-/-/43–60% (*n* = 15)			Lederman 2003 (USA; 2001–2002) [43]
	2/>8/60.8% (*n* = 240)	4/>4/–60.4% (*n* = 240)			Jones 2004 (US; 1999–2002) [40]
	>8/>8/63.8% (*n* = 260)	4/8/64.2% (*n* = 260)		0.06/0.12/- (*n* = 260)	Pfaller 2017 (USA and Europe; 2014) [45]
				0.06/0.12/9.1% (*n* = 366) ^^	Pfaller 2020 (USA and Europe; 2016–2018) [13]
				0.06/0.12/0% (*n* = 152) +	Pfaller 2018 (USA and Europe; 2016) [42]
				0.06/0.12/1.8% (*n* = 112) *	Huband 2019 (USA and Europe; 2017) [44]
				0.06/0.12/2.9% (*n* = 1466)	Pfaller 2017 (North America, Europe, Asia-Pacific, Latin America; 2010–2011) [45]
*H.influenzae*	≤4/≤4/0% (*n* = 199)				Doern 2005 (USA; years 2002–2003) [10]
	-/-/1.4% (*n* = 829)				Melo Cristino 2013 (Portugal; 2003–2004) [38]
	0.5/1/1.4% (*n* = 1172)				Sader 2018 (Europe, Asia, Latin America; 2015–2017) [39]
	2/32/- (*n* = 53)	0.5/4/- (*n* = 53)		1/2/- (*n* = 53)	Macone 2014 (USA) [41]
				0.5/1/0.4% (*n* = 1886) ^	Pfaller 2020 (USA and Europe; 2016–2018) [13]
				0.5/1/0.2% (*n* = 556) **	Huband 2019 (USA and Europe; 2017) [44]
				1/1/0.7% (*n* = 3383) ^	Pfaller 2017 (North America, Europe, Asia-Pacific, Latin America; 2010–2011) [45]
*H. parainfluenzae*	0.5/0.5/4.3% (*n* = 23)				Sader 2018 (Europe, Asia, Latin America; 2015–2017) [39]
				1/2/7.0% (*n* = 71) ^	Pfaller 2020 (USA and Europe; 2016–2018) [13]
				1/-/16.7% (*n* = 6)	Huband 2019 (USA and Europe; 2017) [44]
*M. catarrhalis*	-/-/1.0% (*n* = 303)				Melo Cristino 2013 (Portugal; 2003–2004) [38]
	0.25/0.5/0.7% (*n* = 613)				Sader 2018 (Europe, Asia, Latin America; 2015–2017) [39]
				0.12/0.25/- (*n* = 984)	Pfaller 2020 (USA and Europe; 2016–2018) [13]
				0.25/0/25/- (*n* = 408)	Pfaller 2018 (USA and Europe; 2016) [42]
				≤0.12/0.25/- (*n* = 313)	Huband 2019 (USA and Europe; 2017) [44]
				0.12/0.25/- (*n* = 1226)	Pfaller 2017 (North America, Europe, Asia-Pacific, Latin America; 2010–2011) [45]
Other *Moraxella* species				0.5/1/- (*n* = 9)	Pfaller 2020 (USA and Europe; 2016–2018) [13]
				0.25/-/- (*n* = 3)	Huband 2019 (USA and Europe; 2017) [44]
*C. pneumoniae*		0.125/0.125/- (*n* = 15)		0.06/0.25/- (*n* = 15)	Karlowsky 2019 [46]
*M. pneumoniae*	0.5/0.5/- (*n* = 20)	0.25/0.5/- (*n* = 20)		0.125/0.25/- (*n* = 20)	Waites 2016 (USA and China) [47]
	0.5/1/- (*n* = 50) ++	0.12/0.25/- (*n* = 10) ++			Waites 2017 (USA, Europe and China) [48]
*L. pneumophila*		1/1/- (*n* = 100)		0.25/0.25/- (*n* = 100)	Dubois 2020 (Canada; years 1995–2004) [49]

Current Clinical Laboratory Standards Institute (CLSI) susceptibility breakpoints for tetracycline and doxycycline against *S. pneumoniae* are ≤1 mcg/mL and ≤0.25 mcg/mL, respectively (zone diameters ≥ 28 mm for both. The *S. pneumoniae* CLSI breakpoints for tetracycline were updated in 2013 (previously ≤2/4/≥8 mcg/mL for susceptible, intermediate and resistance, respectively). The Food and Drug Administration (FDA)-assigned omadacycline susceptibility breakpoint is ≤0.12 mcg/mL [33]. NS, non-susceptible; -, not reported; * minimum inhibitory concentration (MIC)90 and MIC50 were the same for tetracycline-resistant (*n* = 213) and macrolide-resistant strains (*n* = 320); ** MIC90 and MIC50 were the same for tetracycline-resistant strains (*n* = 132); ^ includes both beta-lactamase and non-beta-lactamase producing strains; ^^ MIC90 and MIC50 were the same for tetracycline-resistant strains (*n* = 657); + MIC90 and MIC50 were the same for macrolide-resistant (*n* = 413) and tetracycline-resistant strains (*n* = 263); ++ MIC90 and MIC50 were the same for macrolide-resistant strains.

**Table 2:** Doxycycline, minocycline, and omadacycline dosing recommendations for adult outpatients with CAP.

Antibiotic	Administration Route Loading Dose	Administration Route: Maintenance Dose	Special Consideration
Doxycycline [53]	200 mg PO on day 1	100 mg PO every 12 h [7]	Absorption is impaired by antacids containing aluminum, calcium, or magnesium, bismuth subsalicylate, zinc, and iron-containing preparations Patients who are on anticoagulant therapy may require downward adjustment of their anticoagulant dosage May render oral contraceptives less effective Barbiturates, carbamazepine and phenytoin decrease the half-life of doxycycline
Minocycline [54]	200 mg PO on day 1	100 mg PO every 12 h or 50 mg PO 4 times a day	Absorption is impaired by antacids containing aluminum, calcium, or magnesium, bismuth subsalicylate, zinc, and iron-containing preparations Patients who are on anticoagulant therapy may require downward adjustment of their anticoagulant dosage May render oral contraceptives less effective Current data are insufficient to determine if a dosage adjustment is warranted in patients with renal impairment (creatinine clearance < 80 mL/min)
Omadacycline [8]	Day 1: 200 mg by intravenous infusion over 60 min OR 100 mg by intravenous infusion over 30 min twice	100 mg by intravenous infusion over 30 min once daily OR 300 mg PO once daily	Patients are required to fast for at least 4 h and then take omadacycline tablets with water. After oral dosing, no food or drink (except water) is to be consumed for 2 h and no dairy products, antacids, or multivitamins for 4 h Patients who are on anticoagulant therapy may require downward adjustment of their anticoagulant dosage

**Table 3:** Mean pharmacokinetic parameters of tetracycline derivatives after a single dose in healthy adult subjects [8,30,51,55,56,57,58,59,60,61,62,63,64,65,66,67,68,69,70].

Drug	Dose & Route of Administration	*C*_max_ (mg/L)	*t*_max_^a^ (h)	AUC_∝_ (mg∙h/L)	CL or CL/*F* (L/h)	CLR (L/h)	*V* or V/*F* (L)	t_½_ (h)	Protein Binding (%)
Doxycycline	100 mg IV	2.80	1	NR	NR	NR	NR	NR	NR
	200 mg IV	4.30–9.30	1	89.8–206 ^b^	2.24–2.32	0.85–0.98	50.5–52.6	13.8–16.2	NR
	100 mg oral	1.70–5.10	1–4	37.4–75.7	2.92–3.16	1.86–2.1	64.8–92.0	13.6–15.4	NR
	200 mg oral	2.61–5.92	2.6–4.3	85.0–108	1.74–2.90	1.1–3.6	52.6–124	8.8–26.2	82–93
Minocycline	200 mg IV	1.89	NR	25.9	8.21	NR	148	13.7	NR
	150 mg oral	2.10–2.19	2–4	NR	NR	NR	NR	16	76
	200 mg oral	3.10–3.60	2–4	68.6–71.3	4.40	0.5	84	12.9–17.0	NR
Omadacycline	100 mg IV	1.51	0.55	9.36 ^b^	11.2	2.4–3.3	256	16.2	21
	300 mg oral	0.55	2.5	9.40 ^b^	34.6	NR	794	15.0	NR
	450 mg oral	0.87	2.5	8.98 ^b^	NR	NR	NR	13.5	NR

AUC_∝_ area under the plasma concentration–time curve from time zero to infinity, CL clearance for IV administration, CL/F apparent clearance for oral administration, CL_R_ renal clearance, *C_max_* maximum plasma concentration, *IV* intravenous, *NR* not reported, *t*_½_ elimination half-life, *t_max_* time to *C*_max_, *V* volume of distribution, *V*/*F* apparent volume of distribution for oral administration. ^a^ Median value reported. ^b^ Area under the plasma concentration–time curve from time zero to 12 h. Reprinted by permission from (Springer Nature Customer Service Centre GmbH): (Springer Nature) (Clinical Pharmacokinetics (2020) 59:409–425) (Omadacycline: A Review of the Clinical Pharmacokinetics and Pharmacodynamics, Keith A. Rodvold, Rodrigo M. Burgos, Xing Tan, Manjunath P. Pai), (© Springer Nature Switzerland AG 2019) (Published online: 27 November 2019).

**Table 4:** Randomized clinical trials that included doxycycline or omadacycline in adult patients with CAP.

Reference	Study Design and Population	Key Baseline Characteristics	Comparators	Key Primary Clinical Outcomes	Major Findings	Other Findings and Comments
Harazim, 1987 [92]	Double-blind, randomized trial of adult patients with lower respiratory tract infections (*n* = 230)	Of the 230 patients, 219 were assessed for effectiveness: 131 had CAP and 88 had exacerbations of chronic obstructive pulmonary disease (COPD)	Doxycycline 100 mg PO twice daily for 10 days vs. ofloxacin 200 mg or 400 mg PO twice daily for 10 days	Clinical response (cure or improvement) in CAP, defined as disappearance of cough and sputum production	Satisfactory response in CAP patients was 90.0% in the doxycycline group vs. 96.8% in the ofloxacin group 7 patients with CAP in the doxycycline group failed to respond vs. 2 patients with CAP in the ofloxacin group	Overall, 7 patients in the doxycycline group withdrew due to adverse events (gastrointestinal side effects and allergy) vs. 1 patient in the ofloxacin group No *p* values provided
Biermann, 1988 [93]	Double-blind, randomized trial of adult patients with CAP and acute exacerbations of chronic bronchitis (*n* = 221)	Of the 221 enrolled patients, 191 were evaluated (104 with CAP and 87 with exacerbations of COPD)	Doxycycline 200 mg PO daily for 1 day, then 100 mg PO daily for 8 days vs. spiramycin 1000 mg PO three times daily for 1 day, then 1000 mg tablets PO twice daily for 4.5 days	Clinical cure 10–14 days after the start of treatment	In the CAP group, the clinical cure rate was 84.0% with 20% side effects; there was no difference between treatment groups Clinical cure rates for each treatment group with CAP were not specified	Side effects occurred in 21.4% of patients in the doxycycline group vs. 22.5% of patients in the spiramycin group. Most side effects in each group were gastrointestinal 1 patient in the doxycycline group withdrew because of side effects (feeling unwell and blurred vision). 1 CAP patient in the spiramycin group withdrew due to lack of efficacy
Wiesner, 1993 [94]	Double-blind trial of patients with ambulatory respiratory tract infections (*n* = 297)	Bronchitis (*n* = 243), CAP (*n* = 25) and other (*n* = 29))	Doxycycline 100 mg PO once daily vs. erythromycin acistrate 400 mg PO twice daily. The duration of treatment varied from 7 to 14 days depending on severity of infection	Clinical response	Overall, 97.2% of the doxycycline-treated patients improved vs. 96.6% of the erythromycin-treated patients Of the 13 doxycycline-treated patients with CAP, 12 were deemed cured. Of the 11 erythromycin-treated patients with CAP, 9 were deemed cures (*p* values not provided)	Side effects were reported in 16 doxycycline vs. 20 erythromycin patients that were predominantly gastrointestinal in nature Drug discontinuation occurred in 5 doxycycline patients and 3 erythromycin patients
Norrby, 1997 [95]	Double-blind trial of adult inpatients and outpatients with CAP (*n* = 411)	An etiology was confirmed in 270 (66%) cases: 93 had mixed etiology, 133 had by *Mycoplasma* spp., *Chlamydia* spp. or *Legionella* spp., 35 had *S. pneumoniae*, and 30 had a viral infection	Doxycycline 100 mg PO twice daily for 10 days vs. fleroxacin 400 mg PO once daily for 10 days	Clinical response at 2–8 days after end of treatment (first follow up visit) and 3–5 weeks after end of treatment (second follow up visit) in intention-to treat population Clinical response at second follow up visit in per-protocol population	In intention-to treat analyses, clinical response rates in doxycycline- vs. fleroxacin-treated patients were 93% (177/191) vs. 86% (157/182) at first follow up visit and 85% (162/190) vs. 75% (137/182; difference 10%, lower bound for one-sided 95% confidence interval-17.3%) at second follow up visit In the per-protocol analysis, clinical response rates in doxycycline- vs. fleroxacin-treated patients were 92% (162/190 vs. 84% (137/182; difference 7.6%, lower bound for one-sided 95% confidence interval −17.8%) at second follow up visit Null hypothesis that fleroxacin at worst was 15% inferior to doxycycline was rejected for the second follow up visit	Among all pathogens with *S. pneumoniae*, 88% (15/17) of doxycycline-treated patients and 61% (11/18) of fleroxacin-treated patients had clinical success at second follow up visit 12/56 (21%) patients in with Mycoplasma etiology in the fleroxacin group vs. 3/42 (7%) in the doxycycline group were failures at second follow up visit Drug-related adverse events were reported in 39% of 204 fleroxacin patients and in 34% of 207 doxycycline patients (*p* > 0.05) Photosensitivity was the most frequently reported side effect in the doxycycline arm, but no cases resulted in discontinuation of therapy *p* values not provided
Ailani, 1999 [96]	Unblinded, randomized trial of adult hospitalized patients with mild to moderately severe CAP (*n* = 87)	*S. pneumoniae* was the most frequently isolated (8 in doxycycline group vs. 9 in the control group) 3 doxycycline patients and 3 in control group had *S. pneumoniae* bloodstream infection	Doxycycline IV twice daily vs. other undefined antibiotics chosen at the discretion of the admitting physician (control group)	Time to clinical response and hospital length of stay	Median time to clinical response in doxycycline group vs. the control group was 2 days vs. 4 days; *p* = 0.001 Median hospital length of stay in doxycycline group vs. the control group was 4 days vs. 6 days; *p* = 0.04	Three patients in the doxycycline group required a change in treatment because of lack of response vs. 5 patients in control group All patients with pneumococcal bacteremia recovered 6 doxycycline patients vs. 11 patients in control group had adverse events
Mokabberi, 2010 [97]	Randomized, double-blind trial of adults hospitalized patients with CAP (*n* = 66)	Baseline pathogen etiology not reported	Doxycycline 100 mg IV/PO twice daily vs. levofloxacin 500 mg IV/PO once daily; first dose was IV	Hospital length of stay Time to change from IV to PO	The mean hospital length of stay was 4 days for doxycycline and 6 days for levofloxacin (*p* < 0.0012) The mean time to change from IV to PO was 2.88 days for doxycycline and 2.73 for levofloxacin	Treatment efficacy not significantly different (*p* = 0.844) 1 failure resulting in antibiotic change in doxycycline group vs. 2 failures in levofloxacin group No side effects were observed in doxycycline group vs. 2 in levofloxacin group
Stets, 2019 [98]	Phase 3 multinational, double-blind, double-dummy non-inferiority trial of adult patients with CABP (*n* = 774)	Adults with CABP and Pneumonia Severity Index risk classes II-–IV CAP pathogens were identified in 49.9% of patients in the intention-to-treat population; *M. pneumoniae* (33%), *S. pneumoniae* (20%), *L. pneumophila* (19%), *C. pneumoniae* (15%), and *H. influenzae* (12%)	Omadacycline 100 mg IV every 12 h for two doses, then 100 mg IV every 24 h vs. moxifloxacin 400 mg IV every 24 h A transition to oral omadacycline 300 PO mg every 24 h or moxifloxacin 400 PO mg every 24 h was allowed after 3 days Total treatment duration was 7 to 14 days	Early clinical response, defined as survival with improvement in two of the four primary pneumonia symptoms (cough, sputum production, pleuritic chest pain, and dyspnea) with no worsening in symptoms at 72–120 h after initial dose Investigator-assessed clinical response at the post-treatment evaluation (5 to 10 days after the last dose), defined as survival with resolution or improvement in signs and symptoms of infection to the extent that further antibacterial therapy was unnecessary	In the intention-to-treat population, omadacycline was noninferior to moxifloxacin with regard to early clinical response (81.1% and 82.7%, respectively; difference, −1.6 percentage points, 95% confidence interval −7.1 to 3.8) Investigator-assessed clinical response rates at the post-treatment evaluation were 87.6% for omadacycline and 85.1% for moxifloxacin (87.6% and 85.1%, respectively; difference, 2.5 percentage points; 95% confidence interval −2.4 to 7.4)	Adverse events occurred in 41.1% of omadacycline-treated patients vs. 48.5% of moxifloxacin-treated patients The most frequent events were gastrointestinal (10.2% and 18.0%, respectively) *Clostridioides difficile* infections occurred in no patients who received omadacycline versus 8 patients (2%) who received moxifloxacin 8 deaths (2%) occurred in the omadacycline group vs. 4 (1%) in the moxifloxacin group
